# Effects of Corrosion on Compressive Arch Action and Catenary Action of RC Frames to Resist Progressive Collapse Based on Numerical Analysis

**DOI:** 10.3390/ma14102662

**Published:** 2021-05-19

**Authors:** Lu Zhang, Tingyu Wei, Hongyu Li, Jian Zeng, Xiaofang Deng

**Affiliations:** 1 College of Civil Engineering and Architecture, Guilin University of Technology, Guilin 541004, China; zhanglu@glut.edu.cn; 2 Department of Management Engineering, Guangxi Polytechnic of Construction, Nanning 530000, China; weitingyujx@sina.com; 3 Nanning City Investment Group Modern Industrial Park Development Co., Ltd., Nanning 530200, China; zengjian1982@yahoo.com

**Keywords:** progressive collapse, corrosion, compressive arch action, tensile catenary action, FEM

## Abstract

Many negative factors can influence the progressive collapse resistance of reinforced concrete (RC) frame structures. One of the most important factors is the corrosion of rebar within the structure. With increasing severity of corrosion, the duration, robustness, and mechanical performance can be greatly impaired. One specific side effect of rebar corrosion is the significant loss of protection against progressive collapse. In order to quantify the effects of rebar corrosion on load-resisting mechanisms (compressive arch action (CAA) and tensile catenary action (TCA)) of RC frames, a series of numerical investigations were carried out in this paper. The previous experimental results reported in the literature provide a benchmark for progressive collapse behavior as a sound condition and validate the proposed numerical model. Furthermore, based on the verified numerical model, the CAA and TCA with increasing corrosion and an elapsed time from 0 to 70 years are investigated. Comparing with the conventional empirical model, the proposed numerical model has shown the ability and feasibility in predicting the collapse resistance capacity in structures with corroded rebar. Therefore, this numerical modeling strategy provides comprehensive insights into the change of load-resisting mechanisms in these structures, which can be beneficial for optimizing the design.

## 1. Introduction

Progressive collapse is a procedure where a primary structural component fails, leading to the failure of the adjoining structural system due to the damage or failure of a vertical load-resisting component such as column, or wall; eventually, the disproportionate collapse or total collapse of the building happens. Today, the reinforcing concrete (RC) building is one of the most commonly used structural systems because of its flexural spacing arrangement, low self-weight, integrity, and its excellent load-resisting behavior. However, some unexpected events may still cause one or more load-bearing members to fail. This is especially important for columns, since the self-weight or any other loading will be transferred to the ground through these members. Many progressive collapse tragedies are caused by column failure, such as the World Trade Center in New York, USA [1]. After an explosion and intense fire, a sequence of columns failed and eventually led to the collapse of the entire building. A department store building collapsed in Sampoong, Korea, in 1995 because a supporting column on the fifth floor lost most of its bearing capacity due to poor construction quality control [2]. From these events, the load-resisting mechanisms of RC frames due to failure in columns certainly warrant the attention of researchers.

**Figure 1:**
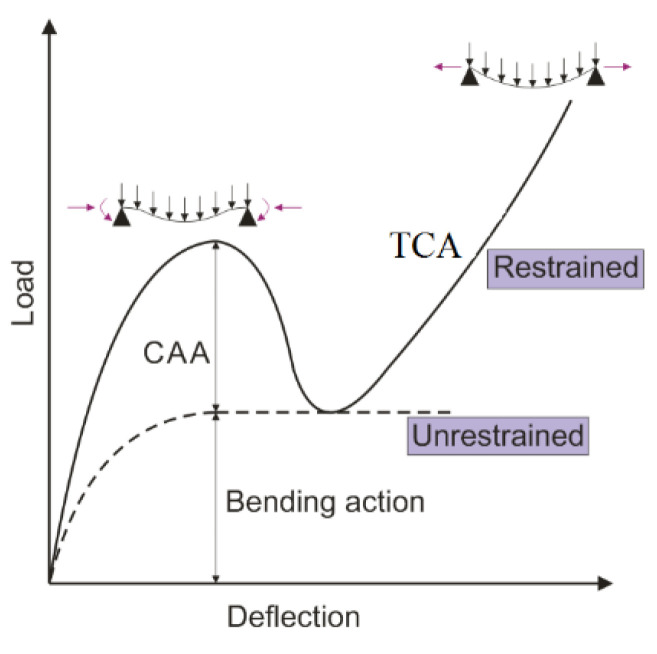
Response curves [14].

There are many researchers currently focusing on the field of progressive collapse. Orton and Sarah [3] investigated the dynamic response of a RC frame with removing column. Four drop tests were conducted to simulate the different loads. The resistant loss happening in the compressive arch and catenary stage can lead to amplify the dynamic effect. Stinger and Orton [4] evaluated the collapse resistance experimentally. They tested a series of RC frames under the column removal scenario and concluded that both compressive arch and catenary action can provide considerable resistance during the collapse. Sasani et al. [5] conducted an experiment on a 10-story RC building after the removal of a column via explosion. When analyzing the collapse behavior, Vierendeel action was considered to be the main representation of force transmission. Lew et al. [6] conducted monotonically increasing vertical displacement on two full-scale RC beam-column assemblies and found that the ultimate load is mostly resisted through the development of catenary action under a column removal scenario. Qian and Li [7,8,9] performed several case studies on a full-scale RC structure. The influence of the joint connection after the removal of a column was observed. It concluded that the interior joints beyond the failed column can provide a support for the beam, which increases the resistance of collapse. Yi et al. [10] experimentally studied the typical resistance forces such as Vierendeel mechanism, arch, and catenary actions which occur during progressive collapse after removal of a column. They found that compressive arch action (CAA) can significantly enhance the load-carrying resistance of RC structures. Li et al. [11,12] investigated the progressive collapse behavior of full-scale RC beam-column joints after the removal of a side column. The performance of beam-column joints was assessed in terms of force transmission. It was observed that severe shear failure happened in the joint due to Vierendeel action. Yu and Tan [13] conducted the push-down test on beam-column sub-assemblages with strong constraints at beam ends. The beam with strong horizontal constraints developed considerable CAA and TCA which can effectively enhance the flexural capacity, eventually improving the progressive collapse behavior, see Figure 1. As shown in this figure, the behavior of CAA and TCA is illustrated in terms of the relationship between load and deflection. Both of them can increase the capacity because of the effect of restraint.

As reported in the previously mentioned literature, lots of academic efforts related to the experimental works have been made. Through experimental study, the load-resisting mechanisms of RC frames (e.g., Vierendeel action, CAA, TCA, and membrane actions) were characterized and the empirical models are summarized accordingly. However, the small-scale tests still have many limitations, such as scale effect, repeatability, and expansibility; meanwhile, full-scale tests are very costly and still have inevitable limitations. Thus, the experimental results from laboratory tests are usually more qualitive. In order to extend the experimental results, the numerical method is a good alternative. With the validated numerical model, the actions occurring in progressive collapse can be characterized quantitively. Evolution of the involved actions can be identified with multiple variables. Therefore, the numerical method has various successful applications for understanding the behavior of RC frames to resist progressive collapse [15,16,17,18,19]. Most of the numerical studies are concentrated on the different actions which may mobilize during collapse. For example, re-distribution of stress and strain and the degradation of strength and stiffness are evaluated directly. The factors which can lead to increased resistance are revealed by parametric study. With the numerical model, more detailed and quantitative analysis becomes possible.

Though the load-resisting mechanisms of progressive collapse have been extensively studied in the literature numerically and experimentally, to take account of the corrosion into the numerical or empirical model is still challenging. Current research on the corrosion in RC buildings typically starts with a partial member in the RC building; additionally, the strength decrease in the rebar or concrete is often the target. For example, Shayanfar et al. [20] found that the reduction in concrete compressive strength is directly related to the corrosion. Biondini et al. [21,22] investigated the seismic resilience of RC frames, including the effects of corrosion. Their results show that the reduction of shear strength and displacement ductility due to corrosion will influence the seismic performance of RC frames. Similarly, Berto et al. [23] discussed the relationship between the load-carrying capacity and corrosion in rebar. Considering the progress of corrosion, the time-dependent variation of safety and serviceability in a single component can be evaluated.

Though many studies have shown that corrosion negatively affects the performance of RC buildings in regard to strength and dynamics performance, the correlation between corrosion and progressive collapse resistance is still vague. Consequently, this paper proposed to understand the characteristics of CAA and TCA during the collapse based on the numerical model. The numerical model was validated by comparing to the experimental results from the push-down test. The correlation of resistance mechanisms and corrosion has been described. The aging process was simulated in the model in terms of the material and mechanical degradation of core concrete, cover layer, rebar, elongation, and bond slip. The proposed numerical model can effectively predict the CAA and TCA behavior, which can be used to mitigate progressive collapse in structures. The structure of this paper is as follows: The brief introduction of progressive collapse and corrosion study is presented in this section, the experiment is described in Section 2, the numerical model and validation are presented in Section 3, the effects of corrosion on CAA and TCA are analyzed in Section 4, and finally, the discussions and conclusions are presented in Section 5 and Section 6, respectively.

## 2. Materials and Methods

In this study, the experimental results from our group’s previous works [23] are to build the benchmark, which can be used for characterizing the behaviors of progressive collapse without corrosion. Additionally, the experimental data can also be used for verifying the numerical model. The validated numerical model can also be extended to consider the varying degree of corrosion. As the first step, the experimental configuration and the corresponding data from [24] were adopted in this section. Further study of the influence of corrosion can be based on this prototype study. The influence of corrosion is studied, accordingly.

### 2.1. Experimental Samples and Configuration

The prototype structure is a 4 × 4 panels, single-story frame. This is the same frame studied by Qian et al. [24]. However, in this study, the substructures with 2 × 2 panels excluding RC slabs were chosen for simplification. The selection of the simplified subassembly in this case is mainly to help focus on CAA and TCA. Usually, this type of substructure with at least two spans allows those actions to develop. Therefore, two types of specimen labeled as T- and P- were fabricated. The details of dimension and reinforcement are summarized in Table 1. Herein, T1 and T2 are the three-dimensional beam-column structures with different combinations of beams, in which P1 and P2 can be considered as the longitudinal and transverse beam of T1, respectively. The differences between T1 and T2 specimens are shown in Table 1, which shows the cross-sectional dimension and span of the beam.

**Figure 2:**
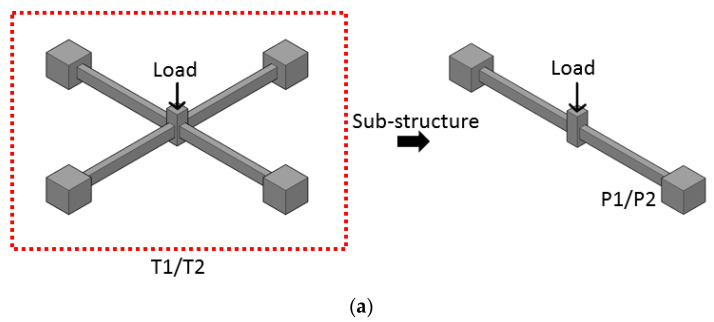

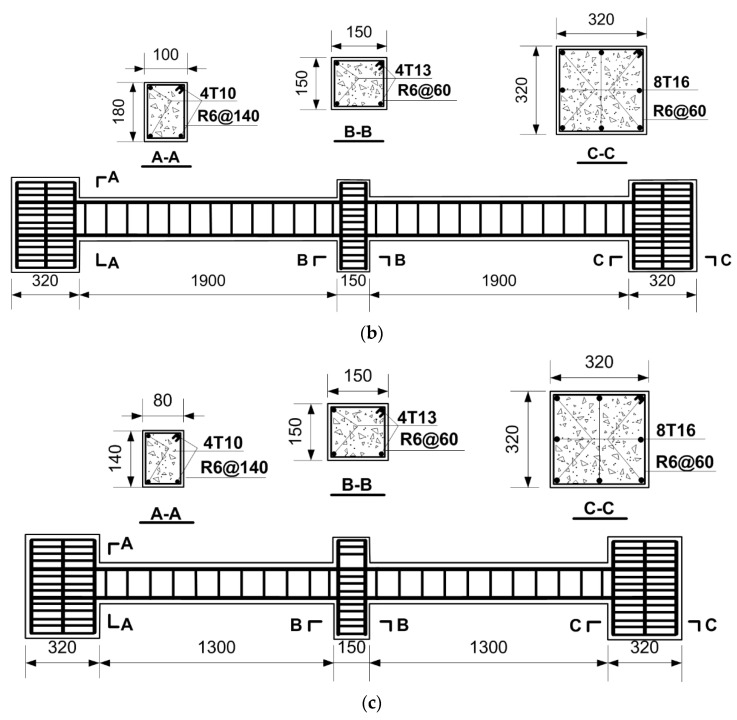
(**a**) Overview of T1/T2 and P1/P2, (**b**) dimension and reinforcement details of P1, (**c**) dimension and reinforcement details of P2 (unit: mm).

The 2 × 2 panel subassemblies consist of two longitudinal and transverse beams and five columns. Taking T1 as an example, the layout and its reinforcement details are shown in Figure 2. As shown in the figure, the longitudinal and transverse beams are labeled as P1 and P2. Since T2 has a similar configuration, the specific figure will not be repeated here—the detailed information can be found in Table 1.

### 2.2. Validation of Numerical Modeling

**Figure 3:**
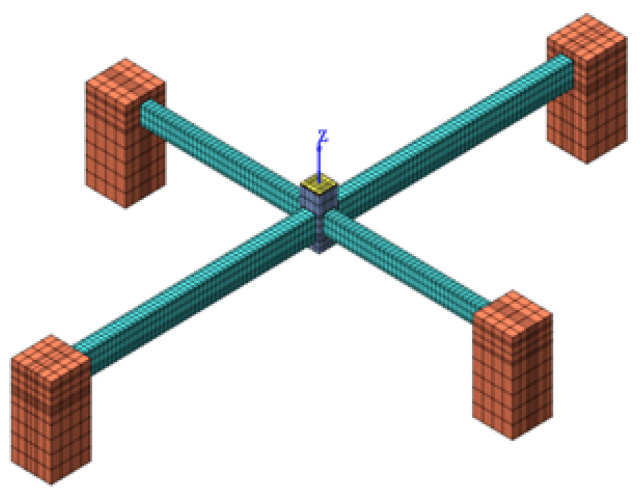
The numerical model of T1.

Nonlinear finite-element analyses of the four selected specimens (T1, T2, P1, and P2) were carried out using the commercial software DIANA 10.4. The push-down test was simulated as follows: In the model, the ground center column is assumed to be removed before analysis. A push-down process is carried out by imposing a prescribed displacement to the top of the interior column, seen Figure 3. To simulate the large deformation response, a total Lagrangian formulation is modeled in DIANA to consider geometric nonlinearity effects. Beams, columns, and beam-column joints are modeled using 8-node solid brick elements (DIANA HX24L element). Compressive behavior is modeled using a parabolic curve based on fracture energy, according to Feenstra [25], and tensile behavior is modeled using a linear tension-softening model based on ultimate strain. Reduction of the compressive strength due to cracks, which is proposed by Vecchio and Collins [26], is used to simulate the cracked concrete in DIANA. Reinforcing bars are modeled by truss elements (DIANA L2TRU element). The Von Mises plasticity model is also applied. Bond-slip between the reinforcement element and the surrounding concrete element is modeled by combining a line interface element (DIANA L8IF element) and a bond-slip relationship proposed by Dörr [27]. For the boundary conditions, a fixed support is assumed at the two side columns. Though the fixed boundary condition would slightly overestimate CAA, the development of TCA associated with large deformation in the beams could be better predicted this way [28]. As an example, the FE model and its mesh of T1 are illustrated in Figure 3. Moreover, the material properties of concrete and reinforcement used in the numerical simulation are listed in Table 2 and Table 3, respectively.

**Figure 4:**
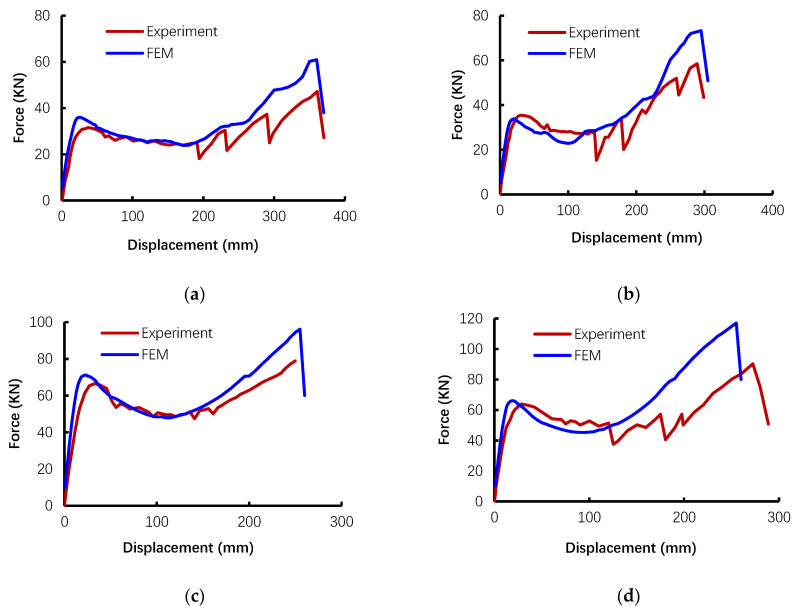
Comparison of the load–displacement relationship of test specimens: (**a**) specimen P1, (**b**) specimen P2, (**c**) specimen T1, (**d**) specimen T2.

Figure 4 compares the load–displacement relationship from finite-element (FE) simulations and tests. As shown in the figure, the overall trends between numerical and experimental results agree well with each other. However, differences can still be observed. Specifically, the evaluation of proposed FE models and specific differences between them are summarized in Table 4. The ratio of numerical results to the experimental measurement associated with the corresponding coefficient of variation (COV) was used to evaluate the accuracy of the FE models. In general, the FEM can reproduce the yield strength well with a mean ratio of 0.99 and a COV of 0.06. For the first peak load referring to the CAA stage, the numerical results are slightly overestimated with a mean ratio of 1.02. This overestimate can be attributed to stronger boundary conditions in the simulation. The possible fixed-end rotation due to bar slips is ignored in the simulation, leading to overestimating the stiffness of the boundary condition. Additionally, the ultimate loading capacity of the subassembly obtained from simulation is at most 30% higher than that from the experiment (cases P1 and T2). The enlarged values of beam-column connection strength are mainly due to the failed elements that were still working in the model, when in reality they would be removed. As shown in Figure 4, based on the proposed model, obvious TCA can be observed and affects the post-peak load-deflection behavior. The proposed geometrical nonlinear model can also successfully predict the softening phase of the curve, which was caused by concrete crushing. However, the subsequent increase in strength due to activation of TCA changed. It is clearly concluded that the numerical model can properly capture the enhancing effect of CAA and predict the TCA with suitable accuracy. In addition, it can provide a validated modeling approach for further corrosion-related analysis.

## 3. Modeling in RC Structures under Corrosion

### 3.1. Overview of the Modeling Model

The FE model has been verified by comparison with the experimental results. Though some differences between the numerical and experimental results can still be observed, the load-resisting mechanisms (TCA and CAA) can be properly reproduced by the FE model. Therefore, the FEM is extended to investigate the influence of corrosion. Rebar corrosion can negatively affect many aspects of a RC structure, including: (i) steel cross-section reduction, leading to the decay of load-resisting capacity, (ii) the change of mechanical properties of the reinforcing bar, including steel elongation and ductility [29], and (iii) corrosion products generating along the rebar, which will cause spalling and crack the concrete cover. In the FEM, those effects can be considered and formulated as six models: diffusion process, reduction of the cross-section of reinforcing bars, degradation of steel mechanical properties, loss of concrete cover, reduction of the confinement in core concrete, and the deteriorating bond of corroded bars [30,31]. In this section, the mathematical models embedded in the FEM are introduced. In general, diffusion process, reduction of cross-section of reinforcing bars, degradation of steel mechanical properties, and loss of concrete cover are usually taken into account in the numerical model. The descriptions of these four models have been presented in detail in our group’s previous work [32]. In this section, only the core equations are introduced:

1. For the diffusion process, the initial time of diffusion process is the concern. In order to obtain the starting time of corrosion, Fick’s second law has been used for characterizing the activities of chloride ions in the concrete. The measured variables, environmental condition, and uncertainties of specimen can be simultaneously considered by the probabilistic model [33]. The initial time of diffusion can be solved by the first-order second-moment (FOSM) method for reliability analysis [34]. All of the related parameters and the specific values are obtained based on the statistical analysis in the literature [33,35].
2. Section reduction of reinforcing bars is one of the most critical variables which can cause decay in the load-resisting mechanism. In order to consider the section loss, we assume the section area as constant before corrosion. The reduction in the rebar section initiates with the corrosion. Associated with the initial time of diffusion process, the progress of reduction of rebar can be given by [35]:
  (1) $$D_{i}\left(t\right)=\{\begin{array}{c} D_{i}\;\;\;\;\;\;\;\;\;\;\;\;\;\;\;\;\;\;\;\;\;\;\;\;\;\;\;\;\;\;\;\;\;\;\;\;t\leq T_{corr}\\ D_{i}-\alpha {\left(t-T_{corr}\right)}^{0.71}\;\;\;\;\;\;\;\;\;\;\;T_{corr}\leq t\leq T_{f}\\ 0\;\;\;\;\;\;\;\;\;\;\;\;\;\;\;\;\;\;\;\;\;\;\;\;\;\;\;\;\;\;\;\;\;\;\;\;\;\;\;t>T_{f} \end{array}$$
  where, $D_{i}$ is the dimeter of rebar without corrosion, t is the time, and $\;T_{corr}\;\mathrm{and}\;T_{f}$ are the corrosion initial time and the time when the diameter becomes zero due to corrosion, respectively. *α* is the deterioration factor.
3. The degradation of steel mechanical properties is mainly reflected in the strength and ductility of the rebar. The yield strength, ultimate tensile strength, and elongation of rebar with the consideration of corrosion can be calculated based on Cairns’ theory [36]. The corrosion-induced reduction in yield strength, ultimate tensile strength, and elongation can be calculated, accordingly. The specific calculation will not be repeated here. The corrosion-induced reductions from 10 to 70 years are summarized in Table 5.
4. With the increase of corrosion, the corrosion products can cause the cracking and spalling of concrete cover. To be specific, the loss of concrete cover is considered by reducing the concrete strength. The reduction of concrete strength can be calculated according to Coronelli and Gambarova’s model [37]. In the model, only corrosion in the beams is considered because the beam is the main member which can provide the alternative load path with removal of the column. The residual strength of concrete cover with time of 10 to 70 years is summarized in Table 6.
5. It has also been observed that corrosion of stirrups leads to the degradation of confined performance of core concrete [38]. Many studies have shown that the contribution of corrosion stirrups to the bearing capacity and ductility of beams [39,40,41] or columns [42,43,44] is reduced due to the corroded stirrups, which fails in confining the core concrete. Therefore, the weakening effect of confinement to the core concrete due to corroded stirrups should also be considered in numerical simulation [37]. The stress–strain relationship of confined concrete proposed by Saatcioglu et al. [45] is introduced and calculated as follows:
  (2) $$f_{cc}=f_{co}+k_{1}f_{le}$$
  where $f_{cc}$ and $f_{co}$ are confined and unconfined strengths of concrete, respectively. $f_{l}$ is the lateral pressure and $f_{le}$ is the equivalent uniform pressure, which is calculated as:
  (3) $$f_{le}=k_{2}f_{l}$$

The coefficients $k_{1}$ and $k_{2}$ are the functions of the lateral pressure, $f_{l}$, and the stirrup characteristic value, $\lambda_{v}$, respectively. According to the model by Qi et al. [46], $k_{1}$ and $k_{2}$ are obtained from regression analysis of experimental data:

(4) $$k_{1}=4.122{\left(f_{l}/f_{co}\right)}^{-0.1}$$

(5) $$k_{2}=0.511{\left(b/a\right)}^{0.88}\lambda_{v}{}^{-0.2}$$

where a and b are the short and long sides of the section, respectively. The lateral pressure, which is approximately uniform, is expressed by:

(6) $$f_{l}=\frac{\sum A_{s}f_{ys}}{b_{c}s}$$

where $A_{s}$ and $f_{ys}$ are the area and yield strength of transverse reinforcement, respectively. $b_{c}$ is perimeter center-to-center of hoops and s is stirrup spacing. Since the stirrups are responsible for large amounts of confinement, the possible corrosion expansion of stirrups and the delamination of the core concrete will cause a significant decrease in core concrete strength. Stirrup corrosion is taken into account by using the relation proposed by Niu et al. [47], and stirrup characteristic value, $\lambda_{v}$, is modified by multiplying a degradation coefficient (δ) as follows:

(7) $$\lambda_{v}^{\prime }=\delta \lambda_{v}=\left(1-1.077\eta_{s}\right)\lambda_{v}$$

where $\eta_{s}$ is the corrosion rate of steel. Therefore, the coefficient $k_{2}^{\prime }$ referred to coefficient determined by stirrups, is adopted in the study:

(8) $$k_{2}^{\prime }=0.511{\left(b/a\right)}^{0.88}{\left(1-1.077\eta_{s}\right)}^{-0.2}\lambda_{v}{}^{-0.2}$$

By substituting Equations (4), (6), and (8) into Equation (1), the reduction of the confinement in the core concrete can be finally expressed as follows:

(9) $$\frac{f_{cc}}{f_{co}}=1+1.13{\left(b/a\right)}^{0.08}{\left(1-1.077\eta_{s}\right)}^{-0.2}\lambda_{v}^{0.7}$$

(6) Corrosion of steel rebar could significantly influence the bond in reinforced concrete, especially when severe corrosion happens in the embedded steel bars [48]. Steel rebar corrosion mainly leads to volumetric expansion of corrosion products, which is responsible for the expansive radial pressure at the steel–concrete interface. The splitting bond strength of uncorroded and corroded specimens is defined as $\tau_{cr}$ and $\tau_{cr}^{*}$ respectively, and the ultimate bond strength of uncorroded and corroded specimens is defined as $\tau_{u}$ and $\tau_{u}^{*}$, respectively. Then, the relative bond strength is defined as:

(10) $$\Phi_{cr}=\frac{\tau_{cr}^{*}}{\tau_{cr}}$$

(11) $$\Phi_{u}=\frac{\tau_{u}^{*}}{\tau_{u}}$$

where, $\Phi_{cr}$ and $\Phi_{u}$ are the relative bond strength of splitting bond strength and ultimate bond strength, respectively. In general, with the increase of corrosion, the relative bond strength decreases. The degradation models are affected by many factors, such as the concrete tensile strength, concrete cover thickness, and the corrosion rate of steel. $\Phi_{cr}$ and $\Phi_{u}$ are usually determined by experimental analysis. Many studies in the literature [49,50,51] show that the bond strength can be considered as a function of corrosion with different variables. The model proposed by Yuan et al. [52] with simple mathematical expressions is adopted in this study:

(12) $$\Phi_{cr}=1.0-K_{cr}\eta$$

(13) $$\Phi_{u}=1.0-K_{u}\eta$$

where, η is the corrosion rate of steel and $K_{cr}$ and $K_{u}$ are the degradation coefficients of splitting strength and ultimate strength determined by the ratio of concrete cover to the steel diameter (*c*/*d*), which can be expressed using the equations as follows:

(14) $$K_{cr}=12.397-3.021\left(c/d\right)$$

(15) $$K_{u}=10.544-1.586\left(c/d\right)$$

Since *c*/*d* has no obvious influence on the slip, the slip can be expressed by the following formula, which only considers the influence of corrosion rate of steel, $\eta_{s}$:

(16) $$s_{cr}^{*}=\left(1.0-5.984\eta_{s}\right)s_{cr}$$

(17) $$s_{u}^{*}=\left(1.0-7.365\eta_{s}\right)s_{u}$$

where, $s_{cr}$ and $s_{cr}^{*}$ are the corresponding slip at splitting bond strength of uncorroded and corroded specimens, respectively. $s_{u}$ and $s_{u}^{*}$ are the corresponding slip at ultimate bond strength of uncorroded and corroded specimens, respectively.

### 3.2. Accommodation of the Corrosion Models

**Figure 5:**
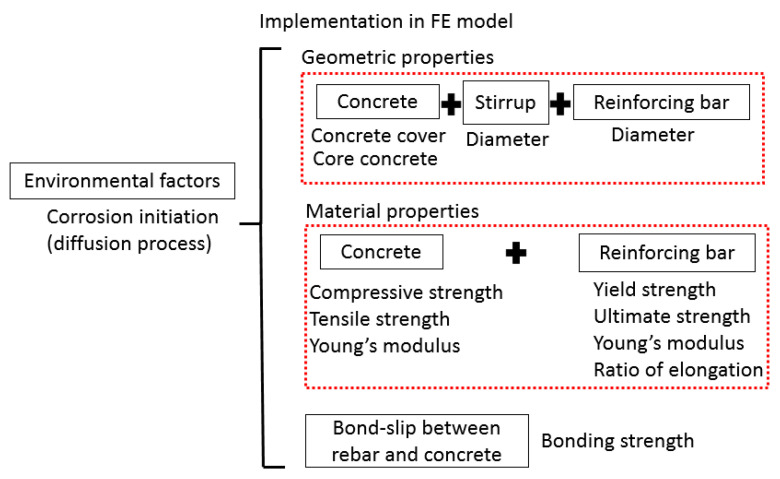
The implementation of corrosion models.

The FE model without consideration of corrosion has been verified in Section 2.2. In addition, six main corrosion leading models are stated in Section 3.1. To accommodate the corrosion models in the numerical simulation becomes the main concern. The process of deploying six models is illustrated in Figure 5. As shown in this figure, the starting time of corrosion is determined by the Fick’s second law. Before the starting time, no corrosion is considered in the model. The corrosion leading degradation reflects on three aspects in the model: (i) geometric properties change, mainly including the diameter of stirrup and rebar and concrete cover and volume, (ii) material properties decay, and the concrete and rebar’s material properties due to different levels of corrosion can be calculated and input into the numerical model, and (iii) the interaction between reinforcing bar and concrete is simulated by changing the bonding strength.

## 4. Results

**Figure 6:**
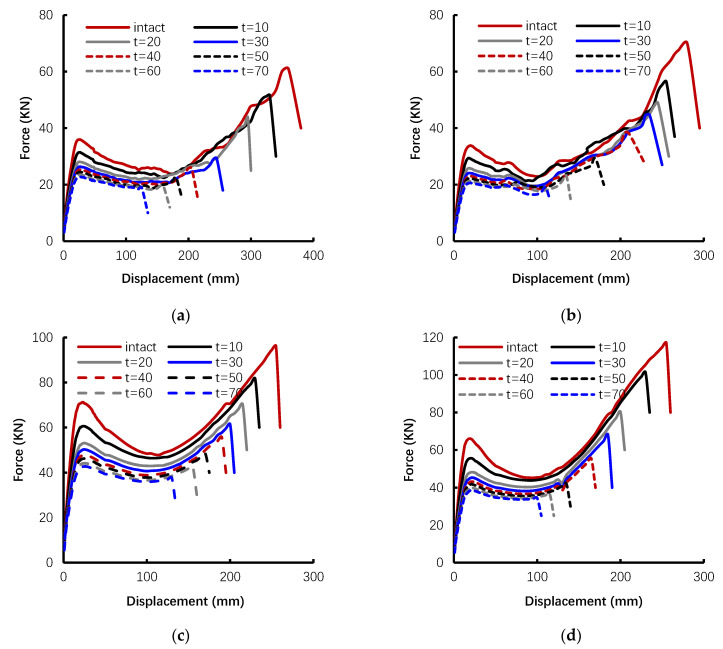
Push-down curves for intact and corroded specimen at t = 10 to 70 years: (**a**) specimen P1, (**b**) specimen P2, (**c**) specimen T1, (**d**) specimen T2.

According to the proposed corrosion models in Section 3, the push-down responses of the four subassemblies (T1, T2, P1, and P2) are investigated over a 70-year lifetime, starting with the structure in sound condition, then simulating response of different corroded degrees with t = 10 to 70 years and an incremental time of 10 years shows the comparison of the push-down curve of the sub-assemblages obtained from the sound and varying levels of corrosion, see in Figure 6. By increasing the service time, significant reduction of the load capacity and ductility can be observed from the corroded cases with respect to their sound conditions. In order to easily describe the progressive collapse behavior, three loading capacities including yield load ($F_{y}$), the first peak load ($F_{p}$), and the ultimate load ($F_{u}$) are extracted from each push-down curve. The detailed features are summarized in Table 7. Besides the loading capacities, the reduction percentages over time, $r_{y}=(F_{y,t=0}-F_{y,t})/F_{y,t}$, $r_{p}=(F_{p,t=0}-F_{p,t})/F_{p,t}$, and $r_{u}=(F_{u,t=0}-F_{u,t})/F_{u,t}$ are calculated for $F_{u}$, $F_{p}$, and $F_{u}$, respectively. The effects of corrosion on the loading capacities $F_{y}$ and $F_{p}$ are less than that of $F_{u}$. For illustration, taking t = 70 years as an example, in general, with the increase of corrosion, $r_{y}$, $r_{p}$, and $r_{u}$ are shown to decrease, but the corresponding rates of $r_{y}$, $r_{p}$, and $r_{u}$ are different. In particular, $r_{y}$ and $r_{p}$ are between 20% and 40%, while $r_{u}$ is over 70%. The significant decay in $F_{u}$ is mainly attributed to the corrosion-induced degradation of the strength and ductility of reinforcing bars.

Also, as shown in Table 7, the first peak load ratio ($F_{p}/F_{y}$) and the ultimate load ratio ($F_{u}/F_{p}$) are defined in order to evaluate the development of capacity of CAA and TCA in beams, respectively. In all cases, the first peak load ratio ($F_{p}/F_{y}$) is over 1. In the sound condition, the contribution of CAA to the peak loads is between 35% and 58%. However, after 70 years of corrosion, the enhancement of CAA reduces. The corresponding $F_{p}/F_{y}$ lowers down to 9–29%. The decay of CAA contribution can be explained from two aspects: (i) the degradation of concrete cover, the compressive strength of the concrete cover will significantly reduce with corrosion and the value will be less than 2.4 MPa after 70 years, and (ii) the spalling of concrete cover.

For the TCA stage, unlike the limit state of first peak load (CAA), the ultimate load mainly depends on the tensile forces provided by reinforcements. The decay in ultimate load ratio ($F_{u}/F_{p}$) is more severe with respect to the sound condition due to the loss of section area and the ductility of reinforcing bars. In addition, values of $F_{u}/F_{p}$ may drop below 1 with increasing degrees of corrosion. The corroded structure cannot take full advantage of tensile forces in reinforcing bars due to the limitation of ductility, which impairs the development of TCA.

## 5. Discussion

In the previous section, the corrosion influence on progressive collapse behaviors has been investigated by the proposed numerical model, which has been validated experimentally. The main load-resisting mechanisms, CAA and TCA, have been identified. With an increasing degree of corrosion, the CAA and TCA decreased, leading to the reduction of collapse resistance.

Though the numerical model has been validated by comparison with the experimental results, the comparison is conducted using pristine results, which means no corrosion has been considered experimentally. Therefore, it is important to discuss the feasibility of numerical results and predictions. In addition, implementing the corrosion-related contrast test on such large-scale beam-column sub-assemblages is difficult due to the time and cost limitations. However, there are several well-established empirical models, such as Park’s model [53], Wang’s model [54], Zhou’s model [55], Su’s model [56], and Hou’s model [57], which can characterize the CAA and TCA. They are widely used in evaluating the capacity of collapse resistance. However, the corrosion is not considered in those empirical models. Therefore, in this section, firstly, we try to include the corrosion factors proposed in Section 3 in the conventional empirical models. Secondly, we compare the load-resisting capacity in different stages (CAA and TCA) obtained from our numerical analysis with that from the empirical model. Thus, the superiority of numerical analysis over the empirical model can be illustrated. In addition, the proposed numerical approach of evaluating the load-resisting capacity can be further proven to have wide applicability. In this section, Park’s and Wang’s models were used to calculate the ultimate capacity in the CAA stage, and the empirical models proposed by Su and Hou were adapted to calculate the capacity in the TCA stage. The detailed models can be found in the literature [49,50,51,52,53] and will not be discussed in this section.

### 5.1. Consideration of Corrosion Factors in Empirical Models

To embed corrosion into Park’s and Wang’s models, the degradation of rebar and concrete are calculated based on the models introduced in Section 3. Therefore, the corrosion influence on the capacity in the CAA stage can be calculated accordingly. Similarly, for predicting the capacity of TCA, the Su and Hou’s empirical models are introduced. The core equations of empirical models, the corrosion-related variables, and implementations of embedding corrosion are summarized in Table 8. The deteriorations due to the corrosion mainly reflect on the performance degradation in rebar and concrete. Specifically, the corrosion starting time, reduction of rebar section, core strength, and bonding strength are considered in the empirical model. Then, the resistance compacity with the different degree of corrosion can be calculated. Unlike the FE model, the empirical model is based on the experimental observations. In order to build a confident and accurate prediction, the empirical model usually needs very large sample sizes. However, in the applications of civil engineering, especially for RC building, it is very costly. Therefore, those empirical models usually only provide a very general prediction in resistant compacity. Some of the details are ignored, which could bring unexpected error. In the following sections, we will discuss the prediction by comparing the results from numerical and empirical models.

### 5.2. Ultimate Capacity in the CAA Stage

**Figure 7:**
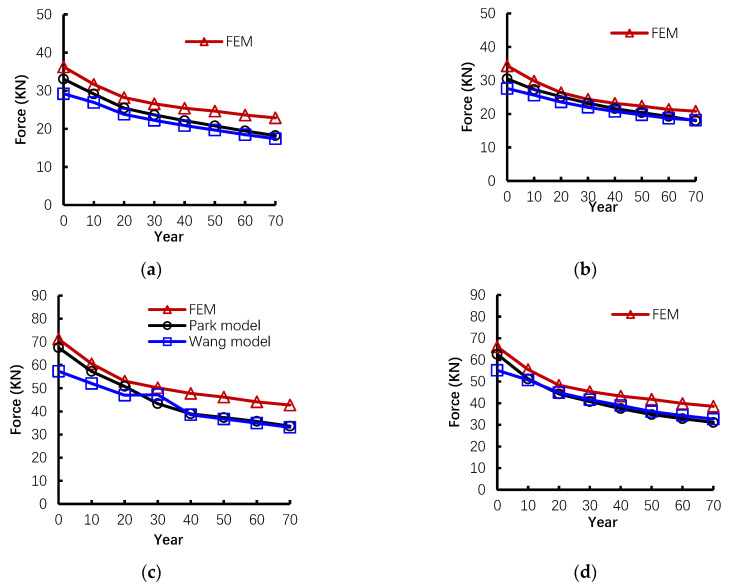
The comparison of first peak load in CAA stage between empirical and numerical results for (**a**) P1, (**b**) P2, (**c**) T1, and (**d**) T2.

The capacity of P1, P2, T1, and T2 from the empirical model and proposed numerical analysis are illustrated and compared in Figure 7. As shown in this figure, for all specimens, the trends of empirical and numerical results agree well with each other. Overall, this means that the proposed method is feasible to predict the capacity in the phase of CAA. However, the proposed numerical model overestimates the capacity compared with the other empirical models because interactions between rebar and concrete are considered in FEM, while they are ignored in the empirical model.

### 5.3. Ultimate Capacity in the TCA Stage

**Figure 8:**
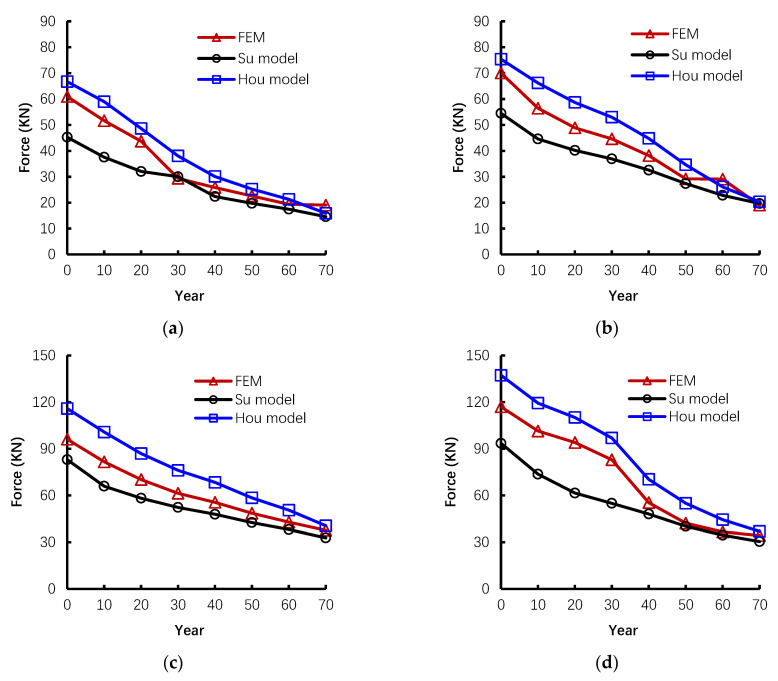
The comparison of ultimate capacity in TCA stage between empirical and numerical results from (**a**) P1, (**b**) P2, (**c**) T1, and (**d**) T2.

For the capacity of TCA, the results of P1, P2, T1, and T2 are compared in Figure 8. Likewise, the numerical results also show similar trends as the empirical results. However, the estimated values lie between Hou’s and Su’s predictions. Usually, the stage of TCA is approaching the failure of the whole structure. The numerical results did not exaggerate the capacity; instead, they can provide a relatively temperate prediction. For solving the capacity of TCA, the key torsion angle is the most critical variable, which directly reflects the behavior of effective height of section and diameter of rebar when corrosion happens. In Su’s model, the torsional angle is taken into account. Meanwhile, the numerical model can reproduce the displacement response. Therefore, the torsion angle can be more accurately predicted. However, Hou’s model simplified the calculation by using an approximate model. The torsion angle is ignored. Therefore, though Hou’s model is simplified, with consideration of torsional angle, the numerical calculation agrees with both Su’s and Hou’s models. It means that the accuracy of numerical simulation highly depends on comprehensiveness of the parameters. The numerical model can provide another option to form the empirical model.

To sum up, from the aspects of estimation of capacity, the proposed numerical prediction in CAA is radical because the capacity is overestimated, which is beneficial for fully taking advantage of resistance from the materials and structure. The temperate prediction in TCA errs on the side of safety. Moreover, the numerical model depends on understanding the physical phenomenon during collapse. Most details, including the material and geometric changes, can be encompassed as long as the proper mathematical models are selected. In addition, the merit of the numerical method is its expandability over the empirical model. With the help of the verified numerical model, it is convenient and cost-effective to conduct the parametric analysis, and also, it can provide a feasible way to consider the potentially influential factors in the numerical model.

## 6. Conclusions

In this work, the influence of rebar corrosion on the progressive collapse behavior of RC buildings, especially for the CAA and TCA stages, has been investigated with a high-fidelity numerical model. In this paper, the progressive collapse is caused by the removal of a column, which is one of the most common scenarios in progressive collapse events. The corrosion is assumed to be chloride-induced, which is a typical hazardous factor on the durability of a structural system. The major aspects of this work are summarized as follows:

1. In regard to the chloride-induced corrosion, six influential factors are introduced to the numerical model with comprehensive consideration of corrosion effects on the rebar and concrete. The mechanisms of each factors were discussed; additionally, the specific implementation of influential factors in the numerical model were presented.
2. With the proposed numerical model, the CAA and TCA have restored. The CAA was weakened gradually with an increasing degree of corrosion. After 70 years of corrosion influence, the ultimate capacity, provided by CAA, decays to 15.6% of that in sound condition. Similarly, corrosion has shown to impair the TCA even more significantly; after 50 years of service, the capacity from TCA is negligible.
3. The process of embedding corrosions in empirical models was provided. The merits of the numerical model over conventional empirical models have been discussed in terms of comparison of the prediction of resistant compacity. Though both methods give similar estimation of capacity, the FE model depends on the instinct nature of decay in material and geometry, which provides a comprehensive insight into the corrosion-induced deterioration in RC buildings. The discrepancy between the empirical and numerical models is due to ignorance of features in the empirical model. In addition, the better expandability of the numerical method can provide a proper guidance for design. The model can be further improved through experimental correction.

## Figures and Tables

**Table 1:** The detailed specimen configuration.

Test ID	Geometrical Properties				Configuration of Reinforcement			
	Beam		Column		Beam		Column	
	Transverse	Longitudinal	Interior	Side	Transverse	Longitudinal	Interior	Side
P1	A	-	C	D	4T10	-	4T13	8T16
P2	-	B	C	D	-	4T10	4T13	8T16
T1	A	B	C	D	4T10	4T10	4T13	8T16
T2	B	B	C	D	4T10	4T10	4T13	8T16

A = clear span = 1900 mm, cross-section = 100 × 180 mm^2^; B = clear span = 1300 mm, cross-section = 80 × 140 mm^2^; C = cross-section = 150 × 150 mm^2^; D = cross-section = 320 × 320 mm^2^; T10 = deformed bar with 10 mm diameter; T13 = deformed bar with 13 mm diameter; T16 = deformed bar with 16 mm diameter.

**Table 2:** Properties of concrete.

Test ID	Compressive Strength $f_{c}^{\prime }\;(\mathrm{MPa})$	Tensile Strength $f_{t}\;(\mathrm{MPa})$	Young’s Modulus $E_{c}\;(\mathrm{GPa})$
P1	19.9	2.22	27.8
P2	20.8	2.28	28.3
T1	21.5	2.33	28.6
T2	22.7	2.42	29.2

**Table 3:** Properties of reinforcement.

Types	Yield Strength $f_{y}\;(\mathrm{MPa})$	Ultimate Strength $f_{u}\;(\mathrm{MPa})$	Young’s Modulus $E_{s}\;(\mathrm{GPa})$	Ratio of Elongation (%)
R6	355	2.22	185.9	17.5
T10	437	2.28	192.2	13.1
T13	535	2.33	205.4	11.6
T16	529	2.42	198.6	14.3

R6 = plain round bar with 6 mm diameter; T10 = deformed bar with 10 mm diameter; T13 = deformed bar with 13 mm diameter; T16 = deformed bar with 16 mm diameter.

**Table 4:** Comparison of the loading capacity of specimens.

Test ID	Yield Load $F_{y}\;(\mathrm{kN})$		Peak Load $F_{p}\;(\mathrm{kN})$		Ultimate Load $F_{u}\;(\mathrm{kN})$		$\frac{F_{y(\mathrm{Test})}}{F_{y(\mathrm{FEM})}}$	$\frac{F_{p(\mathrm{Test})}}{F_{p(\mathrm{FEM})}}$	$\frac{F_{u(\mathrm{Test})}}{F_{u(\mathrm{FEM})}}$
	Test	FEM	Test	FEM	Test	FEM			
P1	24	26.02	32	36.25	47	60.95	1.08	1.13	1.30
P2	26	25.37	36	34.37	59	70.05	0.98	0.95	1.19
T1	48	45.17	67	71.20	79	96.15	0.94	1.06	1.22
T2	48	46.45	64	66.06	90	117.04	0.97	1.03	1.30
						Mean:	0.99	1.06	1.25
						COV:	0.06	0.06	0.04

**Table 5:** Corrosion-induced reduction rate of material properties for the considered corrosion scenarios at *t* = 10 to 70 years.

*t* (Year)	Section Loss (%)	Reduction in Yield/Ultimate Strength (%)	Reduction in Elongation (%)
10	2.5	1.2	7.4
20	7.6	3.8	22.8
30	11.4	5.7	34.2
40	14.7	7.3	44.1
50	17.6	8.8	52.7
60	20.3	10.2	60.9
70	22.8	11.4	68.5

**Table 6:** Corrosion-induced residual capacity of cracked cover concrete at *t* = 10 to 70 years.

*t* (Year)	Reduction in Diameter of Beam Rebar (mm)	Compressive Strength of Concrete Cubes (MPa)			
		P1	P2	T1	T2
0 (intact)	0	24.9	26.0	26.9	28.4
10	0.1	11.9	11.0	13.1	12.3
20	0.4	5.8	5.1	6.3	5.6
30	0.6	4.2	3.6	4.5	4.0
40	0.7	3.4	2.9	3.6	3.2
50	0.8	3.2	2.8	3.5	3.0
60	1.1	2.5	2.2	2.7	2.3
70	1.2	2.2	1.9	2.4	2.1

**Table 7:** Effects of corrosion on loading capacities.

Test ID	t (Year)	$\begin{array}{c} F_{y}\\ \left(\mathrm{kN}\right) \end{array}$	$\begin{array}{c} F_{p}\\ \left(\mathrm{kN}\right) \end{array}$	$\begin{array}{c} F_{u}\\ \left(\mathrm{kN}\right) \end{array}$	$\begin{array}{c} r_{y}\\ \left(\%\right) \end{array}$	$\begin{array}{c} r_{p}\\ \left(\%\right) \end{array}$	$\begin{array}{c} r_{u}\\ \left(\%\right) \end{array}$	$F_{p}/F_{y}$	$F_{u}/F_{p}$
P1	0 (intact)	26.02	36.25	60.95	-	-	-	1.39	1.68
	10	25.24	31.66	51.64	−2.98	−12.68	−15.27	1.25	1.63
	20	25.17	28.20	43.71	−3.27	−22.22	−28.29	1.12	1.55
	30	23.97	26.56	29.35	−7.89	−26.75	−51.84	1.11	1.11
	40	22.99	25.41	25.82	−11.65	−29.91	−57.63	1.11	1.02
	50	22.45	24.66	22.59	−13.70	−31.98	−62.94	1.10	0.92
	60	21.64	23.62	19.38	−16.82	−34.85	−68.20	1.09	0.82
	70	20.99	22.88	19.06	−19.34	−36.90	−68.73	1.09	0.83
P2	0 (intact)	25.37	34.37	70.05	-	-	-	1.35	2.04
	10	24.11	29.82	56.44	−4.98	−13.24	−19.43	1.24	1.89
	20	22.33	26.36	48.88	−12.00	−23.31	−30.22	1.18	1.85
	30	21.09	24.37	44.63	−16.88	−29.11	−36.29	1.16	1.83
	40	20.35	23.18	38.17	−19.81	−32.57	−45.50	1.14	1.65
	50	19.91	22.35	29.17	−21.55	−34.99	−58.36	1.12	1.31
	60	19.27	21.35	23.09	−24.06	−37.88	−67.04	1.11	1.08
	70	18.79	20.83	19.05	−25.96	−39.41	−72.81	1.11	0.91
T1	0 (intact)	45.17	71.20	96.15	-	-	-	1.58	1.35
	10	40.64	60.55	81.64	−10.03	−14.96	−15.09	1.49	1.35
	20	38.06	53.05	70.23	−15.74	−25.48	−26.96	1.39	1.32
	30	37.88	50.22	61.36	−16.15	−29.47	−36.18	1.33	1.22
	40	36.20	47.77	55.59	−19.87	−32.91	−42.18	1.32	1.16
	50	35.48	46.17	48.55	−21.46	−35.16	−49.51	1.30	1.05
	60	33.99	44.11	42.85	−24.75	−38.04	−55.43	1.30	0.97
	70	33.18	42.75	37.63	−26.56	−39.96	−60.86	1.29	0.88
T2	0 (intact)	46.45	66.06	117.04	-	-	-	1.42	1.77
	10	41.04	55.55	101.40	−11.65	−15.92	−13.36	1.35	1.83
	20	39.02	48.28	80.22	−16.01	−26.92	−31.46	1.24	1.66
	30	38.63	45.36	68.06	−16.84	−31.33	−41.85	1.17	1.50
	40	36.96	43.25	55.36	−20.44	−34.53	−52.70	1.17	1.28
	50	36.17	41.81	42.17	−22.14	−36.71	−63.97	1.16	1.01
	60	34.71	39.88	36.61	−25.28	−39.63	−68.72	1.15	0.92
	70	33.75	38.55	34.30	−27.35	−41.64	−70.69	1.14	0.89

**Table 8:** The corrosion embedded into the empirical model.

Empirical Model	Variations Related to the Corrosion	
	Variables	Implementations
Park’s model: $\begin{array}{ll} P & =\frac{1}{\beta l_{n}}\{0.85f_{c}^{\prime }\beta_{1}bh[\frac{h}{2}\left(1-\frac{\beta_{1}}{2}\right)+\frac{\delta }{4}\left(\beta_{1}-3\right)\\ & \;+\frac{\beta l_{n}^{2}}{\delta }\left(\beta_{1}-1\right)\left(\varepsilon +\frac{t}{l_{n}}\right)+\frac{\delta^{2}}{8h}\left(1-\frac{\beta_{1}}{2}\right)\\ & \;+\frac{\beta l_{n}^{2}}{h}\left(1-\frac{\beta_{1}}{2}\right)\left(\varepsilon +\frac{t}{l_{n}}\right)\\ & \;-\frac{\beta_{1}\beta^{2}l_{n}^{4}}{h\delta^{2}}{\left(\varepsilon +\frac{t}{l_{n}}\right)}^{2}]\\ & \;-\frac{{\left(T^{\prime }-T-C_{s}^{\prime }-C_{s}\right)}^{2}}{3.4f_{c}^{\prime }b}\\ & \;+\left(C_{s}^{\prime }+C_{s}\right)\left(\frac{h}{2}-d^{\prime }-\frac{\delta }{2}\right)\\ & \;+\left(T+T^{\prime }\right)\left(d-\frac{h}{2}+\frac{\delta }{2}\right)\} \end{array}$	Rebar section and its tensile strength, Young’s modulus, compression strength of the concrete, vertical displacement due to the failure of middle column	$\varepsilon +\frac{t}{l_{n}}$ is time-dependent, then related variables can be calculated based on the models in Section 3.
Wang’s model: $P=\frac{M_{u1L,y}+M_{u0L,y}}{l_{n1}}+\frac{M_{u1R,y}+M_{u0R,y}}{l_{n2}}\;\;-N\Delta_{u,a}\left(\frac{1}{l_{n1}}+\frac{1}{l_{n2}}\right)-\frac{q}{2}\left(l_{n1}+l_{n2}\right)$	Rebar section and its tensile strength, Young’s modulus, compression strength of concrete, vertical displacement due to the failure of middle column	The axial compression is calculated under the first peak loading. With the axial compression, the stress histogram can be plotted to verify if the obtained stress exceeds the yielding strength until the yield stress is found.
Su’s model: P=2Tsinθ	Rebar diameter, its tensile strength, Young’s modulus, and vertical displacement due to the failure of middle column	Torsion angle is mainly related to the relative position of the midspan, which is controlled by the vertical displacement.
Hou’s model: $P=T_{1}\frac{\left(l_{n1}+l_{n2}\right)\delta }{l_{n1}l_{n2}}$	Rebar diameter	The rebar diameter with different corrosion level is calculated according to the relevant models in Section 3.

## Data Availability

The data presented in this study are available upon request from the corresponding author.
